# Organizational E-Readiness for the Digital Transformation of Primary Healthcare Providers during the COVID-19 Pandemic in Poland

**DOI:** 10.3390/jcm11010133

**Published:** 2021-12-27

**Authors:** Agnieszka Kruszyńska-Fischbach, Sylwia Sysko-Romańczuk, Mateusz Rafalik, Renata Walczak, Magdalena Kludacz-Alessandri

**Affiliations:** 1 Faculty of Management, Warsaw University of Technology, 02-524 Warsaw, Poland; 99999523@pw.edu.pl (A.K.-F.); mateusz.rafalik.dokt@pw.edu.pl (M.R.); 2 Faculty of Civil Engineering, Mechanics and Petrochemistry, Warsaw University of Technology, 09-400 Plock, Poland; 3 College of Economics and Social Sciences, Warsaw University of Technology, 09-400 Plock, Poland

**Keywords:** primary healthcare, COVID-19, organizational e-readiness, digital transformation, technological capabilities, operational capabilities

## Abstract

The COVID-19 pandemic has forced many countries to implement a variety of restrictive measures to prevent it from spreading more widely, including the introduction of medical teleconsultations and the use of various tools in the field of inpatient telemedicine care. Digital technologies provide a wide range of treatment options for patients, and at the same time pose a number of organizational challenges for medical entities. Therefore, the question arises of whether organizations are ready to use modern telemedicine tools during the COVID-19 pandemic. The aim of this article is to examine two factors that impact the level of organizational e-readiness for digital transformation in Polish primary healthcare providers (PHC). The first factor comprises operational capabilities, which are the sum of valuable, scarce, unique, and irreplaceable resources and the ability to use them. The second factor comprises technological capabilities, which determine the adoption and usage of innovative technologies. Contrary to the commonly analyzed impacts of technology on operational capabilities, we state the reverse hypothesis. The verification confirms the significant influence of operational capabilities on technological capabilities. The research is conducted using a questionnaire covering organizational e-readiness for digital transformation prepared by the authors. Out of the 32 items examined, four are related to the operational capabilities and four to the technological capabilities. The result of our evaluation shows that: (i) a basic set of four variables can effectively measure the dimensions of OC, namely the degree of agility, level of process integration, quality of resources, and quality of cooperation; (ii) a basic set of three variables can effectively measure the dimensions of TC, namely adoption and usage of technologies, customer interaction, and process automation; (iii) the empirical results show that OC is on a higher level than TC in Polish PHCs; (iv) the assessment of the relationship between OC and TC reveals a significant influence of operational capabilities on technological capabilities with a structural coefficient of 0.697. We recommend increasing the level of technological capability in PHC providers in order to improve the contact between patients and general practitioners (GPs) via telemedicine in lockdown conditions.

## 1. Introduction

The COVID-19 pandemic has created a need for the urgent digital transformation of various organizations. It has also highlighted many of the challenges facing healthcare systems around the world today and has had a significant impact on the way healthcare entities perform. These changes affect an organization’s operating strategies and decisions. A way to maintain efficiency in such a rapidly changing environment is through operational (OC) and technological capabilities (TC), which are valuable, scarce, unique, and irreplaceable and can be used by healthcare entities to effectively manage the digital transformation [1,2,3].

Information and communication technology (ICT) may contribute to solving many problems and can create new opportunities and challenges in traditional healthcare. ICT has been recognized as an essential tool for enhancing healthcare quality, accessibility, and delivery. Conventional health services have been replaced by remote medical care to ensure continuity of treatment. However, challenges have arisen in the healthcare sector, such as poor integration with multiple levels of care, inadequate sharing of health data, and limited interoperability of clinical applications that hinder digitalization efforts [4].

The evolution, adoption, and high failure nature of health information technology systems require effective organizational e-readiness for digital transformation (organizational e-readiness—OeR) assessments to avert failures while increasing system benefits. ICT is spreading to such an extent that it will soon be challenging to imagine a healthcare entity not using some form of IT solution [5]. To support organizations to start defining their digitalization transformation roadmap, a model to analyze the status quo is needed. Therefore, research on OeR in healthcare is very important [6].

This paper is a part of the ongoing research aiming to examine organizational e-readiness for digital transformation, including the implementation of modern telemedicine tools. In this research, we focus on two dimensions of OeR: operational (OC) and technological capabilities (TC). The main objective of this study is to investigate the levels of these capabilities in Polish primary healthcare entities under the conditions of the COVID-19 pandemic. It was also important to study the impacts of OC on TC.

The introduction of technologies into healthcare organizations is a complex phenomenon [7]. Healthcare organizations are pluralist and heterogeneous systems where change may be contested. OeR for the integration of technology is critical to the success of the digital transformation. This is a multilevel, multifaceted construct [8], which can be broadly defined as a set of general capabilities, including strategic capabilities (clearly articulated goals and values, leadership, value for patients), operational capabilities (resources, processes, collaboration), cultural capabilities (governance, communication, openness to risk), competence capabilities (staffing, skills, training, knowledge), and technological capabilities (automated processes, digital workplaces, digital methods and tools) [9,10,11]. When e-readiness exists, healthcare organizations are more prepared to accept and implement change, and if organizations are not ready, the technology is more likely to be rejected or abandoned [12].

In this paper, we focus on two dimensions of OeR based on capabilities, namely operational capabilities (OC) and technological capabilities (TC). We define OC as the sum of valuable, scarce, unique, and irreplaceable resources and the ability to use them [10,13], involving standardized and integrated processes and routines [10,14] based on agile cooperation between people [10]. This kind of operation supports an ongoing learning mechanism within the organization [15]. In contrast, TC are related to the adoption and usage of innovative technologies with the goal of automating core processes [10,16,17], enhancing coordination within the organization to support information exchange [18,19,20] and ongoing learning processes [21,22], and leveraging motivation and commitment for change [23,24,25] by providing a supportive digital workplace [10].

The publications to date have broadly considered TC and OC in research on OeR for digital transformation, although the key gap visible in the existing literature concerns the impacts of OC on TC. Instead, research on TC shows how managers are able to use technological resources to achieve organizational goals [26] and how technological opportunities affect OC [27,28]. The inverse relation still calls for more research studies.

We assume in this study that the effective implementation of technologies depends on the state of practice, which may slow down or prevent IT implementation. Indeed, a minimum level of OC is required before implementing digital technologies in a company [29]. Our study on how OC impacts TC contributes to the understanding of the organizational e-readiness of primary healthcare providers for digital transformation and how this knowledge can be integrated into a synthetic model. The research findings can help healthcare actors gain a holistic view of the relationship between OC and TC and their impacts on digital transformation e-readiness.

This paper is structured as follows. Section 2 presents a literature review. Section 3 includes the specifications of the applied research methods, including the constructs and variables used and an explanation of the data-gathering process. Section 4 presents the results obtained in the study of the factors in the model of organizational e-readiness for digital transformation. The exploratory factor analysis (EFA) is used to define the factors. This section also includes a confirmatory factor analysis (CFA) in order to investigate the impacts of OC on TC. Section 5 consists of a discussion of the limitations of this study. Finally, the paper also provides conclusions and practical implications.

## 2. Literature Review

OeR research shows that immanent readiness assessment capabilities for digital transformation exist. However, OeR studies are fragmented, with a lack of reliable measuring tools [30]. The analysis of 106 peer-reviewed articles conducted by Weiner et al. revealed conceptual ambiguities and disagreements in the current thinking and writing regarding OeR for change [9]. According to the authors, OeR refers to the extent to which organizational members are psychologically and behaviorally prepared to implement organizational change [9].

Wayne et al. [31] developed a comprehensive assessment of organizational functioning and readiness for change. It focuses on motivation and personality attributes of program leaders and staff, institutional resources, and the organizational climate as an essential first step in understanding organizational factors related to implementing new technologies into a program.

Molla and Licker [32] studied the factors that affect e-commerce adoption in a developing country. The authors proposed two research models, one of them based on perceived OeR. The model had four components: awareness (innovation context), commitment (organizational context), resources (human, business, and technology), and governance (organizational context).

Kwahk and Lee [33] examined the role of readiness for change in the context of enterprise resource planning (ERP) system implementation. The authors explored the role of readiness for change in ERP implementation and its impact on usage intention. They defined OeR by incorporating the technology acceptance model (TAM) and theory of planned behavior (TPB). They included two antecedents of readiness for change (perceived personal competence and organizational commitment) and two process outcome variables (perceived usefulness and perceived ease of use) leading to ERP usage intention. The results showed that readiness for change had an indirect effect on behavioral intention to use an ERP system. At the same time, readiness for change was found to be enhanced by two factors: organizational commitment and perceived personal competence.

Shea’s et al. [34] presented in their article the results of a psychometric assessment of a measure called Organizational Readiness for Implementing Change (ORIC), which was developed based on Weiner’s theory of organizational readiness for change. According to Weiner’s theory, organizational readiness for change is a multilevel and multifaceted construct. It can be assessed at the individual or supra-individual levels (e.g., team, department, or organization). Hence, the implementation of many promising innovations in healthcare requires collective, coordinated actions by many organizational members, and it is justified to focus on the supra-individual level. The two facets of readiness are change commitment and change efficacy. Change commitment reflects organizational members’ shared resolve to implement a change. The second facet of readiness, change effectiveness, reflects organizational members’ shared belief in their collective capabilities to implement a change.

This work is part of research that aims to formulate a framework for the organizational e-readiness of primary healthcare for digital transformation that explains capabilities and management choices necessary to respond to the adoption of ICT solutions in the new post-COVID-19 reality. Among the different constructs that could be used for the e-readiness assessment, two with higher impacts were analyzed in this study, which may be valid for any organization: OC and TC.

We assume in this study that superior performance is based on resources and capabilities that are specific for various organizations, rare, and hard to copy by competitors. Ensuring the continuity of patient care in the era of accelerated adoption of ICT-based solutions on the one hand requires healthcare entities to assess OC in order to use them properly, and on the other hand requires TC that enable effective work with new solutions.

Over the last two decades, TC have been recognized as key organizational capabilities and an important driver of superior performance [35]. ICT technology creates a supporting infrastructure that can streamline and accelerate the activities of digital transformation [36]. As a result, entities with an appropriate level of TC are better prepared for digital transformation to improve operations and continuity planning in crisis situations [37]. TC play an important role in achieving organizational efficiency and a degree of innovativeness. They are associated with the skills and knowledge necessary for an organization to absorb, use, adapt, develop, and transfer technologies [38].

TC are defined in the literature as the ability of an organization to implement, integrate, and use its technological resources to increase the organization’s performance [39]. The TC has also been described as the ability of an individual to design and develop new processes, products, and services; to update knowledge and skills about the physical environment in a unique way; and to transform knowledge into instructions and designs to effectively achieve the desired performance [40]. This includes resources of theoretical knowledge, practical experience, procedures, methods, and physical equipment and devices [3]. Besides, it enables the adaptation and assimilation of new knowledge and techniques [41], and effectively combining different technology streams and mobilizing technological resources to improve efficiency [2,42]. Thanks to proper TC in healthcare, a medical entity can provide medical services in a better and more efficient way that best meets the needs of patients, thereby increasing the availability of medical services and improving treatment efficiency and the continuity and coordination of patient care.

TC may play a key role in enhancing the effectiveness of a healthcare entity in terms of innovation and providing health services. TC are generally related to the knowledge and skills necessary for an individual to develop, use, adapt, absorb, and transfer technology [38]. A company’s technology can be thought of as part of the extensive knowledge, techniques, systems, and tools available to produce, distribute, and use goods and services at the end destination. In medical entities, this is related to the development of telemedicine. The level of TC can determine how an entity can successfully apply digital solutions in crisis operations to design and modify digital solutions; improve the patient care process; promote innovation, collaboration, and mobility for doctors, medical staff, and administrative staff; and implement digital solutions.

Our analytical approach regarding the use of TC to assess the primary healthcare OeR is based on four statements, as presented in Table 1.

On the other hand, the advent of the COVID-19 pandemic requires us to rethink how the health sector can prepare for digital transformation. Central to this preparation is laying the foundations for OC. The key to successfully improving OeR is to stimulate the development of OC to implement technological innovations supporting the patient treatment process and healthcare entity management. Therefore, medical entities must focus on the development of OC to constantly experiment with new technologies in order to maintain the appropriate quality of medical services.

OC represent the ability of an entity to perform the operational processes and procedures required to perform operational activities [45,46]. They relate to the integration and coordination of a complex set of tasks, including the ability to use inputs and resources such as raw materials and labor in the production of products and services [47]. Organizations with proper OC have the ability to perform various tasks and activities efficiently [48]. OC also represent the ability of an organization to deploy, integrate, and use its resources to achieve a specific goal [49]. Moreover, this ability does not count as a full OC until it becomes a routine integrated with organizational processes to the point where it allows “repeatable, reliable performance” [50].

Our analytical approach to the use of OC to assess the primary healthcare OeR is based on four statements, as presented in Table 2.

The development and sustainability of improved OC should be a primary focus of management staff [54]. These aspects include leadership and management, employee development, decision making, the adoption of new methods, process management, and performance management [20]. Earlier studies have shown that OC can be facilitated by appropriate management of processes and performance, but neither study offered a comprehensive view of both aspects.

OC in the digital age stem from the way organizations integrate, achieve, and embed digitalization throughout an entity, processes, and strategy. This requires explicit elements such as resources and practices, but also hidden elements such as know-how, skill sets, and leadership. One of the main capabilities needed for digital transformation is the managerial ability to support the development and implementation of digitalization [55].

## 3. Materials and Methods

### 3.1. Methodology

This paper uses triangulation as the research method. After an analysis of the relevant literature on digital transformation e-readiness was carried out, a survey of managers of primary healthcare (PHC) facilities was conducted and the results were then statistically analyzed. The research was conducted on a representative sample of 371 PHC units across Poland. The following statistical analysis methods were used: descriptive and frequency analysis of the results, exploratory factor analysis (EFA), confirmatory factor analysis (CFA), and regression and variance analysis. The research sample should also be selected according to the research method applied. The total of 371 responses meant that the number of reactions was sufficient to carry out EFA and CFA analyses. Those methods require several times more responses than questions in a survey. The 10:1 ratio of responses to questions is the best option, while 5:1 is satisfactory and 3:1 is acceptable [56]. EFA and CFA analyses were used to extract consistent factors from the research question pool. Then, based on the obtained factors, the regression analysis was performed to identify the impacts of OC on TC.

### 3.2. Population and Data Collection

There are about 21,500 PHC facilities in Poland [57]. In total, 371 medical facilities were randomly selected for the study, which constitutes a representative research sample [58]. One PHC manager from each organization took part in the survey. Data were collected in August and September 2021 on behalf of the Warsaw University of Technology. The CATI method was used. The interviewer contacted the PHC facility managers by telephone and recorded their responses. In total, 371 completed questionnaires were delivered to the researchers. The survey contained 32 questions divided into five areas: strategy, organization, culture, competencies, and technology. For answers, the Likert scale was coded as follows: 1—strongly disagree; 2—disagree somewhat; 3—neither agree nor disagree; 4—agree slightly; 5—strongly agree. Eventually, based on initial EFA analysis, only eight questions were selected for further analysis and two interesting factors, OC and TC, were identified (Table 3). The managers were also asked several questions about the finances and staffing of the clinics under their responsibility. As those questions were voluntary, managers rarely provided their responses.

Both OC and TC were established based on the Rossmann concept [10]. The author defines OC as the sum of the degree of agility (variable V_q2s29), level of process integration (variable II_q2s6), quality of resources (variable II_q2s7), and quality of cooperation (variable II_q2s8).

The TC dimension comprises 4 items: adoption and usage of technologies (variable V_q2s31), customer interaction (variable IV_q2s22), process automation (variable IV_q2s30), and digital workplaces (variable IV_q2s23).

### 3.3. Ethics

The questionnaire was prepared based on the literature. The survey instrument was assessed by the research team of the Warsaw University of Technology. Additionally, the management of the PHC facility CortenMedic was consulted about this questionnaire. The content of the survey and rules for carrying it out were accepted by the Warsaw University of Technology Senate Committee for Professional Ethics. The survey was anonymous. Respondents completed it voluntarily and could withdraw from the survey at any time. The questions were read to the managers of the PHC facilities by telephone. All responses were recorded in the database.

## 4. Results

### 4.1. Data Analysis

**Figure 1:**
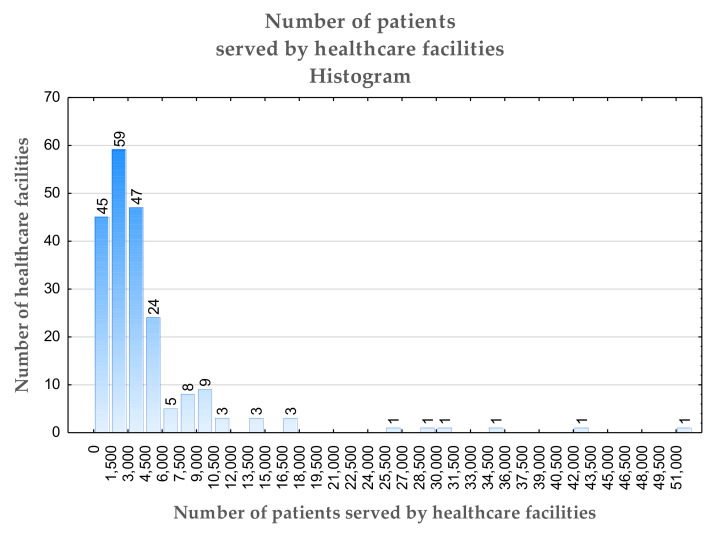
The number of patients served monthly by primary healthcare facilities.

**Figure 2:**
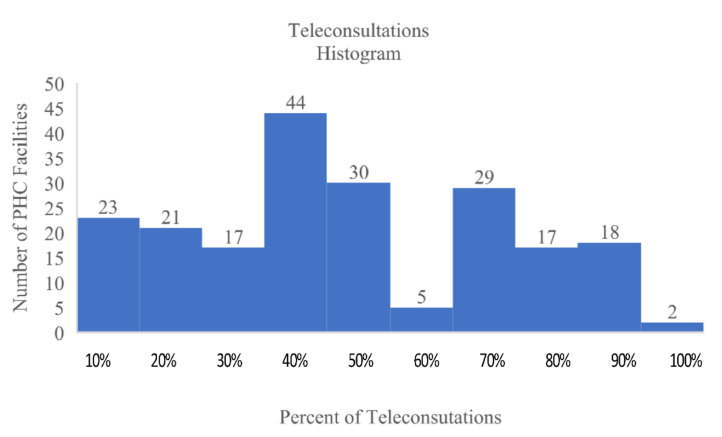
Percentage of teleconsultations provided monthly by primary healthcare facilities.

The survey results were statistically analyzed using IBM SPSS v. 27, IBM AMOS v. 7 (Predictive Solution, Krakow, Poland), and Microsoft Excel 365 software (Microsoft, Redmond, WA, USA). In total, 371 outpatient clinic managers from across Poland answered the survey questions. Sixty-one of the surveyed entities had private owners; the remaining clinics were state-owned. All clinics provided remote consultations during the pandemic. Here, 201 out of 371 managers informed about the number of patients at their facility. The reported sizes of the PHC organizations were mainly small and average (Figure 1). The average number of patients served by the PHC units was 4570; however, the median was equal to 3100. Five big PHC facilities served more than 25,000 patients, while the largest one served more than 42,000 patients. On average, they provided 2017 consultations. The median value was equal to 1200, while the greatest number of visits was 49,116. The share of teleconsultations is presented in Figure 2. On average, this was equal to 35%, with a median value of 33%.

#### 4.1.1. Technological Capabilities (TC)

**Figure 3:**
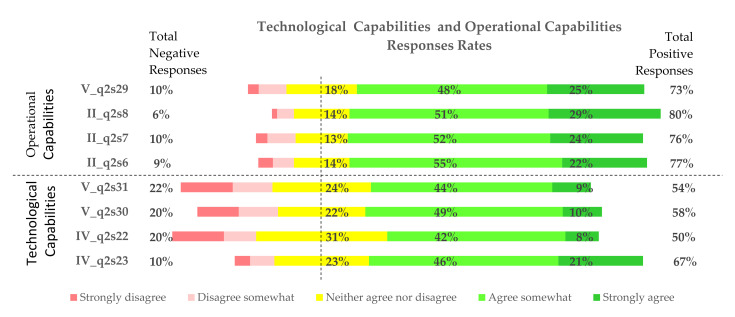
Technological capability and operational capability responses rates.

The TC metrics used in this study relate to the capabilities of healthcare entities to integrate digital technologies such as emerging technologies (e.g., voice interfaces, augmented reality, artificial intelligence, blockchain, etc.), patient experience tools and methods (such as persona and journey maps), digital tools, and modern architectures (APIs, cloud storage, etc.). The variables IV_q2s22, IV_q2s23, V_q2s30, and V_q2s31 were used to assess TC. Descriptive statistics for all variables are presented in Table 4. All distributions were negatively skewed. In each case, more than 50% of respondents positively answered the survey questions; however, there were far fewer responses of “strongly agree” than “agree somewhat”. Considering the answers “neither agree nor disagree” and “agree somewhat”, respondents did not precisely know whether their PHC facilities used new digital tools and technologies to improve medical care. In total, 20% of respondents claimed (variable IV_q2s22: $\bar{x}=3.26$) that their healthcare (HC) units do not use any user experience (UX) tools, while 31% did not know whether they do or not. Additionally, 50% declared that they use new digital instruments, of which 8% understood that this is the case. Furthermore, 10% of PHC managers do not promote innovation, collaboration, and mobility for doctors, medical staff, and administrative staff (variable IV_q2s23: $\bar{x}=3.74$), while 23% did not know whether they do; however, 67% do something about it and 21% strongly confirm innovation activities. Here, 58% of respondents claimed that they use new digital solutions to improve healthcare services (variable V_q2s30: $\bar{x}=3.38$), 22% of managers did not know whether they use any new digital tools, and 20% asserted that they do not use any new digital tools. Respondents answered very similarly to the question V_q2s31 ($\bar{x}=3.38$), with 54% regularly using emerging technologies. However, only 9% strongly confirmed their use, while 22% do not use new tools, 24% did not know about these tools. The distributions of answers are presented in Figure 3.

#### 4.1.2. Operational Capabilities

The OC dimension was assessed based on II_q2s6, II_q2s7, V_q2s29, and II_q2s8 variables. Descriptive statistics are presented in Table 5. As in the case of TC, all distributions are left-skewed. In this case, more than 70% of respondents positively answered all questions. In total, 77% of managers claimed that their organizations defined processes for new digital solutions (variable II-q2s6: $\bar{x}=3.85$), while 22% confirmed that these solutions are ready to use. Only 9% did not define any procedures for digitization. Additionally, 76% of respondents stated that they have all the required resources to implement digital solutions, 24% of PHC facilities have everything ready, and 10% have done nothing to apply IT solutions (variable II-q2s7: $\bar{x}=3.87$). Furthermore, 80% of PHC units (variable II-q2s8: $\bar{x}=4.02$) encourage their staff-doctors, nurses, other medical staff, clerks, and IT specialists to implement digital solutions. Additionally, 73% claimed (variable V_q2s29: $\bar{x}=3.85$) that their procedures are flexible and that they continuously and collectively apply new solutions. All answers to survey questions are presented in Figure 3.

### 4.2. Factor Analysis

#### 4.2.1. Exploratory Factor Analysis

The exploratory factor analysis was performed considering 371 survey responses. Adequacy validity was proven, since all variables in each considered dimension were correlated (Table 6).

All variables presented in Table 3 allowed us to extract two components: OC and TC. Principal axis factoring (PAF) was used as an extraction method; both factors had eigenvalues greater than one. Additionally, Promax rotation with Kaiser normalization was applied. The Kaiser–Meyer–Olkin measure of sampling adequacy (KMO) equaled 0.89 > 0.6. KMO should be greater than 0.6 to accept extracted components as correct. Bartlett’s test of sphericity was significant (χ^2^ = 1309.525; df = 28, *p* < 0.001). Based on the above values, it can be claimed that the sample size is appropriate for factor analysis [59].

The convergent validity of the model was not proven, since the loading of variable IV_q2s23 was smaller than 0.5. The rule of convergent validity requires all variable loadings to be greater than 0.5 and the average value of all loadings to be greater than 0.7 [60]. Once IV_q2s23 was deleted, the condition was fulfilled. Additionally, the discriminant validity of the model, including variable IV_q2s23, was not proven, since this variable loads on two factors and the difference between the loadings was smaller than 0.2. After removal of this variable, the correlation value between two extracted factors was 0.655 < 0.7 [60]. Again, when IV_q2s23 was deleted, the condition of discriminant validity was fulfilled.

**Figure 4:**
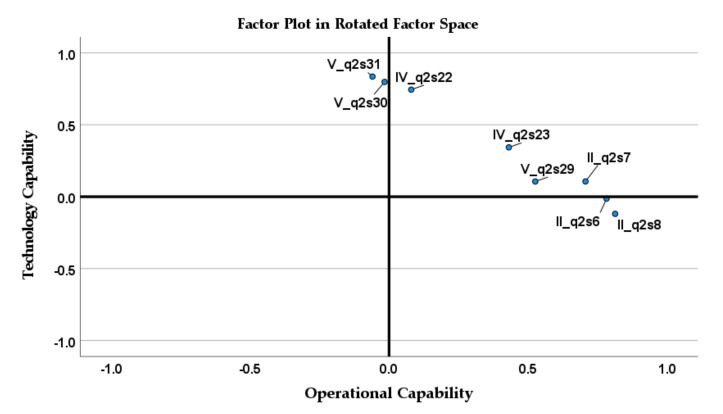
Factor plot in rotated factor space for variables IV_q2s22, IV_q2s23, V_q2s29, V_q2s30 V_q2s31, II_q2s6, II_q2s7, and II_q2s8.

**Figure 5:**
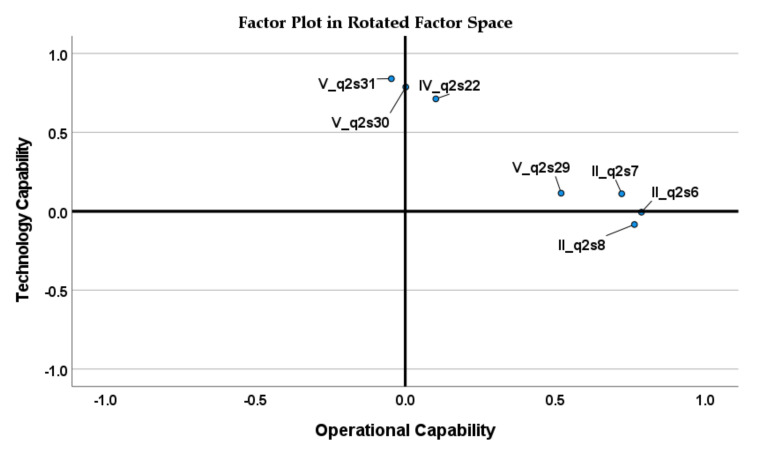
Factor plot in rotated factor space for variables IV_q2s22, V_q2s29, V_q2s30 V_q2s31, II_q2s6, II_q2s7, and II_q2s8.

Considering eight variables, the total variance explained by the model was equal to 56.47% (Table 7). Factor loadings are shown in Table 8. Looking at the factor plot in the rotated factor space in Figure 4, the variable IV_q2s23 does not belong to any factor; it is in the middle of the two-factor groups. Although the Cronbach’s alpha for the TC dimension is greater than 0.7, this variable should be removed from the analysis. The new model, including variables IV_q2s22, V_q2s29, V_q2s30, V_q2s31, II_q2s6, II_q2s7, and II_q2s8, explains 57.36% of the variance. The factor analysis results are presented in Table 9, while new factor loadings are presented in Table 8. Without the variable IV-q2s23, two dimensions can be clearly separated, and it can be argued that all remaining variables represent two extracted dimensions (Figure 5).

#### 4.2.2. Confirmatory Factor Analysis

**Figure 6:**
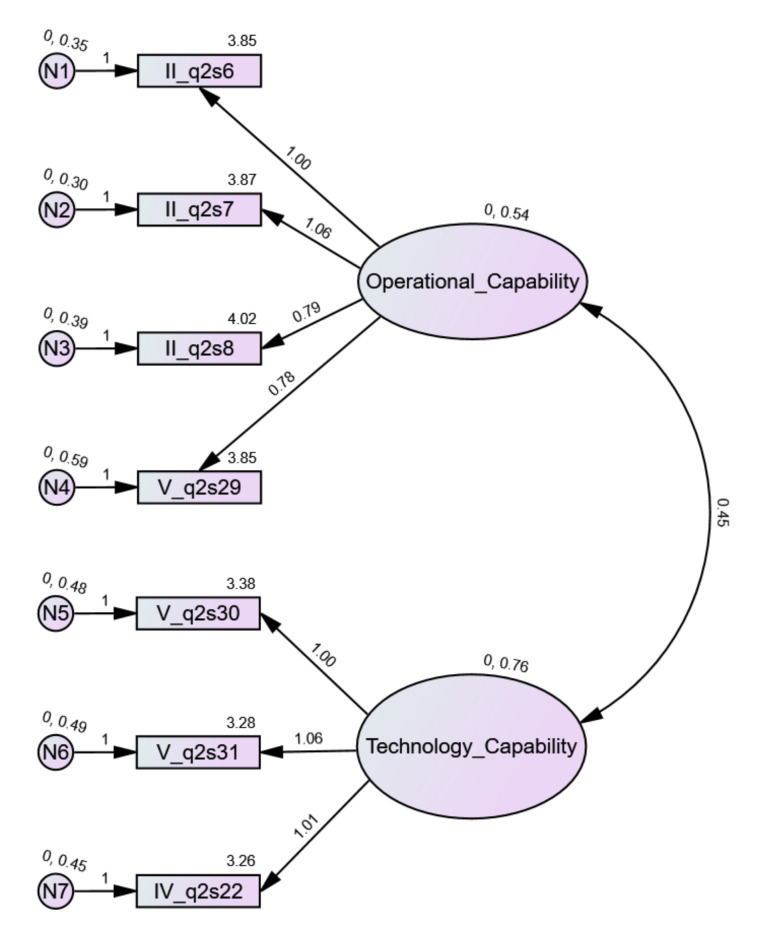
CFA model of organizational e-readiness (unstandardized estimates).

**Figure 7:**
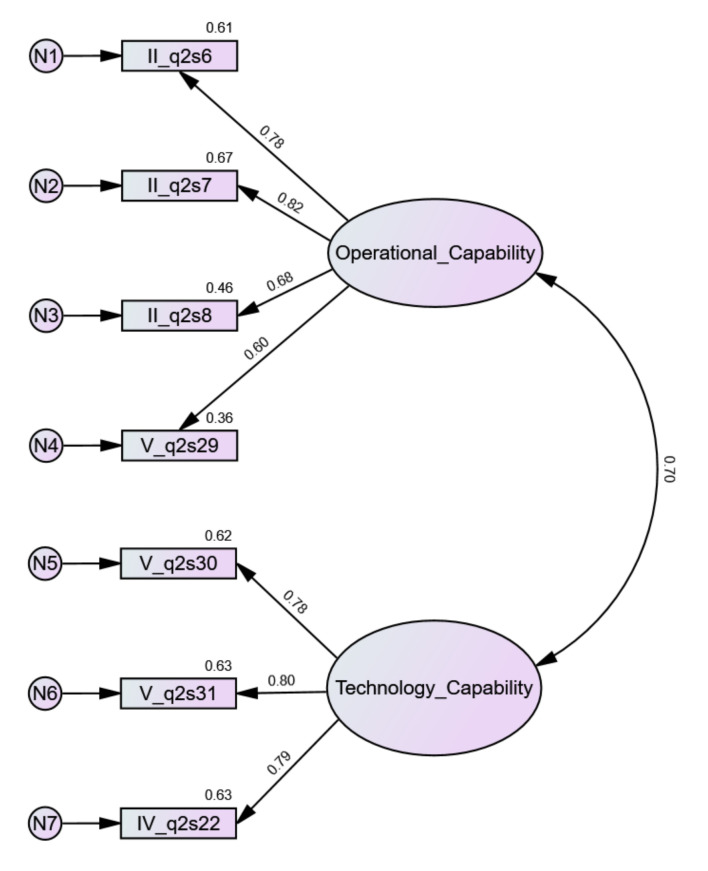
CFA model of organizational e-readiness (standardized estimates).

The assumed model extracted by EFA analysis was confirmed by CFA analysis. Both factors, OC and TC, were correctly extracted. This was supported by the average variance extracted (AVE) values, both of which were greater than 0.5. Additionally, the critical ratio values for both factors were greater than 0.7, which confirmed the convergent validity of the model. The discriminant validity of a model may be confirmed when the square root of the AVE is greater than the correlation between factors. Here, the correlation between OC and TC equaled 0.697 and was smaller than the square roots of the AVE for both dimensions (Table 10). In this way, the discriminant validity of the model was confirmed. The reliability of the model was also confirmed. The chi-square value (Cmin) was equal to 18.806, DF (degrees of freedom) equaled 13. Cmin/DF = 1.447 ∈<1,3>, which was correct. Other measures indicated an excellent model fit: CFI = 0.995 > 0.95; RMSEA = 0.035 < 0.06; SRMR = 0.031 < 0.08; PClose = 0.76 > 0.05. Figure 6 and Figure 7 present unstandardized and standardized solutions for the model.

#### 4.2.3. Regression Analysis

**Figure 8:**
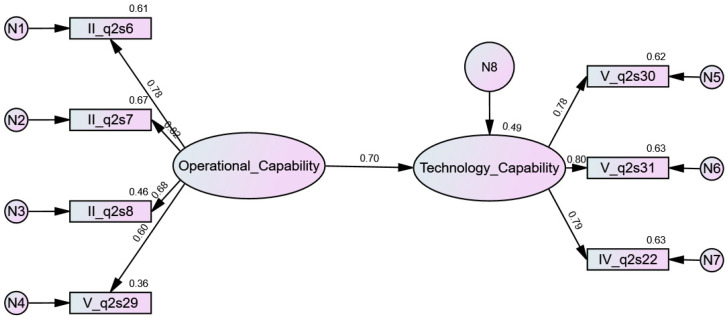
Regression model for organizational e-readiness (standardized estimates).

Regression analysis was performed in IBM SPSS AMOS v. 27.0 software using a structural model (Figure 8). Two hypotheses were formulated:

**Hypotheses** **0** **(H0).** *OC do not influence TC.*

**Hypotheses** **1** **(H1).** *OC do influence TC*.

Based on the structural model, the null hypothesis H0 was rejected in favor of the alternative hypothesis H1. There was a significant influence of the latent factor OC on the second latent factor TC. The structural coefficient on the path between these variables (0.697, Table 11, Figure 8) indicated the rate of change of the dependent variable from the independent variable. The model explained 48.6% of the TC variance. All model fit indices were excellent (Table 12).

## 5. Discussion

Digitization and health services are of great importance within the context of the COVID-19 pandemic, and are expected to become even more important in the future. They significantly impact the way healthcare entities operate and deliver value to patients. Therefore, it is essential to assess the level of organizational e-readiness for digital transformation in individual healthcare entities. There are many specific aspects to consider for evaluation. Digital transformation is the integration of digital technology into an entity’s operational areas to fundamentally change the way the staff operate and the deliver value to the patients.

To understand the constructs of the organizational e-readiness for digital transformation in a healthcare entity, we developed a research model and analyzed it using empirical data from Polish primary healthcare entities. Building on the theoretical underpinnings, this study explores the key OeR factors that drive healthcare adoption of digital technologies. There is discussion in the literature regarding a number of variables that impact OeR, for instance clearly articulated goals and values, leadership, value for patients, organizational culture, governance, communication, openness to risk, competency development, staffing, skills, training, and knowledge [9,10,11]. However, in this paper, we decided to focus on OC and TC, which are of great importance.

We assume that OC depend on defined, well-described, and repeatable processes; adequate resources and the ability to use them; cooperation between physicians, medical staff, and administrative staff; and a flexible, iterative, and collaborative approach to creating digital solutions [17,19,21,22,24,43,44].

The second dimension of OeR in our study is TC that represents the unit’s excellent and heterogeneous technical resources, which are related to design technologies, information and process technologies, and the acquisition and integration of external knowledge [61]. There is discussion in the literature on how to measure TC. In our study, the variables used to measure TC are related to the ability of healthcare entities to integrate emerging digital technologies (e.g., voice interfaces, augmented reality, artificial intelligence, blockchain, etc.), patient experience tools and methods (such as persona and journey maps), and digital tools and modern architectures (APIs, cloud storage, etc.) [13,14,15,38,45,46,47,49,50,51,52,53]. In our study, we assume that technologies should be used by medical entities to improve the patient care process; to design and modify digital solutions; to promote innovation, collaboration, and mobility for doctors, medical staff, and administrative staff; and to promote speed and flexibility in implementing digital solutions. Our variables relate to these purposes.

The main aim of this study was to examine the OeR of Polish primary healthcare providers for digital transformation using two dimensions of organizational e-readiness: OC and TC during the COVID-19 pandemic in Poland. Most of the PHC managers in this study positively assessed both the OC and TC in primary healthcare facilities. This was the case even though telemedicine was never previously used in Polish primary healthcare institutions [62]. According to the results, the PHC managers were rather satisfied with the OC of their entities. Most of them agreed or strongly agreed with all positive aspects of the OC. Most of them confirmed that PHC units encourage their medical, administrative, and IT staff to cooperate in implementing digital solutions (80%) and that their organization has defined processes for new digital solutions (77%). The majority of respondents stated they have all the required resources to implement digital solutions (77%). Earlier studies also confirmed that it is the cooperation of personnel that plays the greatest role in improving OC [63,64]. On the other hand, according to other authors, the ability to implement new digital solutions is a key factor in an OC, as the healthcare industry faces constant demands for change and transformation due to technological progress, market forces, and the regulatory environment [65]. Therefore, it is positive that this aspect was also highly rated by the respondents. The majority of them stated that their procedures are flexible, and that they continuously apply new solutions (73%). High OC allow organizations and employees to learn quickly; identify new resource strategies; integrate, build, and reconfigure internal resources, and have the ability to cope with dynamic environments [26]. The literature emphasizes that the main barriers in improving OC are related to people (about 60%) and resources (about 30%) [66]. Other barriers include low availability [67], inadequate workforce (e.g., high rotation of medical personnel) [68], and lack of cooperation (e.g., potential for conflict) [67] and planning (e.g., no scale-up strategy) [69].

The TC were assessed much worse by the respondents. Only slightly more than half of respondents said that they regularly use new technologies (54%) and new digital solutions to improve health services (58%). All variables for assessing TC were rated below 3.5 on average. Weak TC make it challenging to use telemedicine and have a negative impact on medical care [66]. The barriers related to TC have been confirmed in other studies. Other authors have found that the causes of negative assessments of TC may be differences in equipment, lack of user-friendliness, and outdated systems [70], as well as missing functionalities [71,72] and a lack of usability [73], interoperability [68], human technical support (e.g., lack of skilled workers in IT maintenance) [68], and regional infrastructure (e.g., lack of broadband access) [67].

This study examined the direct relationship between OC and TC. This relationship has been found to be statistically significant. The results show that the use of information technology in a medical entity is mainly dependent on operational factors. The OC are the central part of OeR for digital transformation and significantly improve the technological shown in today’s dynamic health services market. Additionally, some other studies have emphasized that effective OC increases the strength of the TC of the organization. For instance, our conclusions were confirmed in studies conducted in China on the basis of data obtained from 509 companies from various industries [74]. It has been established that OC plays a key role in the implementation of technological innovations. The effective use of OC contributes to the acquisition of new knowledge and skills by entities in order to improve and promote new technologies. Secondly, to improve TC, entities must have specific basic skills, such as the ability to coordinate and the ability to arrange resources to carry out various innovative activities. Therefore, the existing OC are very important for digital transformation and for extending the TC. The literature on modern technology management also suggests that TC depend on other aspects of the organization, such as providing an organizational climate conducive to experimentation and investment in employee development [75]. Unfortunately, the impacts of OC on TC have not yet been the subject of research in healthcare studies.

Most of the studies on OeR have indicated significant positive links between TC and OC. This means that greater use of technology improves unit efficiency both at the operational and organizational level, resulting in better quality of services, cost reductions, and increased overall development [76]. It has been proven that TC improves the OC of entities in organizing activities as well as allocating resources [77]. This is justified by the theory of organizational abilities, according to which the achievements of an organization are influenced by the perspective of OC using digital information technologies. This suggests that TC influence organizational performance through the development of OC, highlighting the role of OC as mediating factors [78]. Technological potential plays a role in shaping higher OC by improving information technology [79]. IT enables effective and efficient processing of information obtained from various sources, which allows entities to increase organizational efficiency [16]. To improve OC, organizations need to have better IT capability, thereby increasing the utilization of available resources [80].

Consequently, technology-oriented entities have the will and ability to acquire crucial technological knowledge and apply it in the process of improving their organization. TC allow improvement of internal processes, which ultimately minimizes the costs of operations and procedures in order to improve efficiency [81]. In the discussion on the impacts of information technology (IT) on organizational performance, it is emphasized that IT creates value for the organization by influencing OC and the organization as a whole [82,83,84].

This article makes several contributions to the literature. Firstly, using the proprietary model to test OeR for digital transformation, we show that this level depends on OC and TC. This discovery will provide guidance for healthcare managers as they develop strategies and decide how to allocate medical resources.

Secondly, until now, TC in the healthcare sector have been studied mainly in the developed countries of Western Europe, the USA, Latin America, and the emerging economies of Asia [85]. Our research on TC in healthcare entities is the first to analyze the situation in this regard in the countries of Central and Eastern Europe.

Finally, to the best of our knowledge, our research is one of the first attempts to study the impacts of OC on TC in healthcare sectors. Most studies in the literature answer the question of how IT helps entities develop higher-level OC. In our model, we investigate the inverse relationship. First, we believe that greater OC improves medical processes and procedures that enable entities to take advantage of new medical technologies needed for remote patient care. Commonly available IT resources cannot be implemented without proper preparation at the operational level [22,86]. Instead, investments in different types of IT must be integrated into operational processes to develop digital transformation e-readiness.

As with any research, this article contains a few limitations that open up possibilities for further exploration in future research. First, our study only focuses on primary care entities, which may limit the possibility of generalizing our results to outpatient entities and hospitals. This survey also looks at the situation in one country only. In the future, it would also be interesting to see what role other organizational and cultural factors play in influencing the relationship between OC and TC, e.g., management heterogeneity, IT staff skills, and organizational structural factors. The research also did not consider the influence of the external context on TC. Another limitation of the study is the bias of data based on subjective opinions of medical personnel. Finally, we did not analyze whether OC and TC have an impact on the development of primary healthcare entities, including the improvement of workflow processes (diagnosis and treatment of diseases) and the improvement of the quality of medical care. The authors of the article have already collected data from the patients and medical staff from the same medical entities on the quality of medical care, which will allow us to study the impacts of OC and TC on related aspects such as continuity, coordination, accessibility, treatment efficiency, and doctor–patient communication.

## 6. Conclusions

In this study, we considered an opportunity-based approach to developing a new model of digital transformation e-readiness. The organizational e-readiness of healthcare entities for digital transformation discussed in this article is an essential topic in relation to the need of patients for remote medical care in the context of the COVID-19 pandemic. In this paper, we focused on two dimensions of organizational e-readiness for digital transformation, OC and TC, as well as the relationship between them.

The results of this study indicate that organizational readiness for digital transformation and digital deployment is influenced by various organizational and technological factors, including provider human resources, IT infrastructure, perceived ease of use of the technology, and provider flexibility. The OC and TC cannot be considered separately. Especially for healthcare providers, the seamless integration of telemedicine technologies into operational processes is important.

Limited TC and OC may prove to be a barrier to the deployment of telehealth in any digital transformation e-readiness model. Organizational constraints, such as labor shortages or limited digital resources, can hinder acceptance and success when implementing telemedicine initiatives. Moreover, telemedicine technology must be developed considering the people involved and the existing processes; it must be adapted to the regional and local infrastructure and accepted by medical staff and patients.

Using various data reduction methodologies, we finally identified a basic set of variables that can effectively measure the dimensions of OC and TC. Following previous studies, four variables were initially selected to measure OC and four variables to measure TC. As a result of a factor analysis, the number of variables used for measuring TC was reduced to three. An exploratory factor analysis showed that the final model adopted for further research was correct. The empirical results showed that OC is on a higher level than TC in Polish PHCs. This may be worrying, as technological potential is one of the driving forces behind digital transformation. Taking into account the significant influence of OC on TC, we strongly argue that primary healthcare entities must apply efficient and effective operational processes to use appropriate IT infrastructure and improve technological opportunities. The research results confirm that the use of OC is a key way to create an environment conducive to the improvement of TC.

In summary, our key findings are as follows:

(i) We developed a new model of organizational e-readiness for digital transformation, based on organizational and technology capabilities;
(ii) A basic set of variables that can effectively measure the dimensions of OC was confirmed and comprised four variables, namely the degree of agility (variable V_q2s29), level of process integration (variable II_q2s6), quality of resources (variable II_q2s7), and quality of cooperation (variable II_q2s8);
(iii) A basic set of variables that can effectively measure the dimensions of TC was reduced to three, comprising the adoption and usage of technologies (variable V_q2s31), customer interaction (variable IV_q2s22), and process automation (variable IV_q2s30);
(iv) The empirical results showed that OC is on a higher level than TC in Polish PHCs;
(v) We have made one of the first attempts to examine the impact of OC on TC in the healthcare sector. Assessment of the relationship between OC and TC revealed a significant influence of operational capabilities on technological capabilities with a structural coefficient of 0.697.

The results of the study will, in a practical way, help managers of PHC assess the e-readiness of their entities for the digital transformation process and overcome any identified barriers in this regard.

## Figures and Tables

**Table 1:** Statements used to assess TC with supporting literature.

Statements	Supporting Literature	Essence
We regularly use emerging technologies (e.g., voice interfaces, augmented reality, artificial intelligence, blockchain, etc.) to improve the patient care process.	Fortuin and Omta (2009) [19]	Strategic capabilities (coordination and information accessibility)
	Ali et al. (2018) [43]	
We use patient experience tools and methods, such as persona and journey maps, to design and modify digital solutions.	Lu et al. (2007) [44]	Indicators of innovation effort process
	Yeniyurt et al. (2019) [17]	
We use digital tools to promote innovation, collaboration, and mobility for doctors, medical staff, and administration.	Jonker et al. (2006) [21]	Learning mechanisms
	Mohamad et al. (2017) [22]	
We use modern architectures (APIs, cloud storage, etc.) to promote speed and flexibility in implementing digital solutions.	Ziggers and Henseler (2009) [24]	Technology upgrade for motivation and commitment to change
	Ali et al. (2018) [43]	
	Li and Chan (2019) [25]	

**Table 2:** Statements used to assess OC with supporting literature.

Statement	Supporting Literature	Essence
We have defined, well-described, and repeatable processes for implementing digital solutions.	Christensen and Overdorf (2000) [14]	Processes and routine
	Wu et al. (2010) [45]	
	Benitez et al. (2018) [51]	
	Helfat and Peteraf (2003) [50]	
We dedicate appropriate resources to the work of digitization.	Coombs and Bierly (2006) [13]	Resources
	Ahmed, Kristal, Pagell (2014) [47]	
	Raphael and Schoemaker (1986) [49]	
Our organizational model encourages collaboration between doctors, medical staff, and administrative staff and IT specialists.	Guan and Ma (2003) [15]	Learning mechanisms
	Bustinza, Molina, and Arias-Ar (2010) [52]	
We have a flexible, iterative, and collaborative approach to developing digital solutions.	De Mori, Batalha, and Alfranca (2016) [38]	Job coordination and contribution
	Kumar and Singh 2019 [53]	

**Table 3:** Survey questions for OC and TC dimensions.

Dimension	Variable Name	Question
Technological capabilities	V_q2s31	We regularly use emerging technologies (e.g., voice interfaces, augmented reality, artificial intelligence, blockchain, etc.) to improve the patient care process.
	IV_q2s22	We use patient experience tools and methods, such as persona and journey maps, to design and modify digital solutions.
	IV_q2s23	We use digital tools to promote innovation, collaboration, and mobility for doctors, medical staff, and administrative staff.
	V_q2s30	We use modern architectures (APIs, cloud storage, etc.) to promote speed and flexibility in implementing digital solutions.
Operational capabilities	II_q2s6	We have defined, well-described, and repeatable processes for implementing digital solutions.
	II_q2s7	We dedicate appropriate resources to the work of digitization.
	II_q2s8	Our organizational model encourages collaboration between doctors, medical staff, and administrative staff and IT specialists.
	V_q2s29	We have a flexible, iterative, and collaborative approach to developing digital solutions.

**Table 4:** Technological capabilities and descriptive statistics from the survey responses.

Variable	Mean	Median	Mode	Std. Deviation	Skewness	Kurtosis	Cronbach’s Alpha
IV_q2s22	3.26	3	4	1.109	−0.711	−0.202	0.841
IV_q2s23	3.74	4	4	0.976	−0.835	0.652	
V_q2s30	3.38	4	4	1.114	−0.815	−0.127	
V_q2s31	3.28	4	4	1.161	−0.687	−0.428	

**Table 5:** Operational capabilities and descriptive statistics from the survey responses.

Variable	Mean	Median	Mode	Std. Deviation	Skewness	Kurtosis	Cronbach’s Alpha
II_q2s6	3.85	4	4	0.947	−1.162	1.522	0.809
II_q2s7	3.87	4	4	0.958	−1.054	1.051	
II_q2s8	4.02	4	4	0.852	−0.984	1.331	
V_q2s29	3.85	4	4	0.962	−0.898	0.680	

**Table 6:** Correlation matrices for OC and TC variables.

OC Variables	II_q2s6	II_q2s7	II_q2s8	V_q2s29
II_q2s6	1	0.643	0.552	0.447
II_q2s7	0.643	1	0.546	0.471
II_q2s8	0.552	0.546	1	0.442
V_q2s29	0.447	0.471	0.442	1
TC variables	IV_q2s22	IV_q2s23	V_q2s30	V_q2s31
IV_q2s22	1	0.568	0.612	0.629
IV_q2s23	0.568	1	0.497	0.466
V_q2s30	0.612	0.497	1	0.64
V_q2s31	0.629	0.466	0.64	1

**Table 7:** Factor analysis results for variables IV_q2s22, IV_q2s23, V_q2s29, V_q2s30, V_q2s31, II_q2s6, II_q2s7, and II_q2s8. Extraction method: principal axis factoring.

Factor	Initial Eigenvalues			Extraction Sums of Squared Loadings			Rotation Sums of Squared Loadings
	Total	% of Variance	Cumulative %	Total	% of Variance	Cumulative %	Total
1	4.313	53.909	53.909	3.886	48.579	48.579	3.418
2	1.039	12.983	66.893	0.631	7.892	56.471	3.310
3	0.609	7.608	74.501				
4	0.549	6.867	81.368				
5	0.445	5.559	86.927				
6	0.375	4.685	91.612				
7	0.348	4.345	95.957				
	0.323	4.043	100.000				

**Table 8:** Factor loadings for TC and OC.

Variable	Factor IV_q2s23 Included		Factor IV_q2s23 Deleted	
	Operational Capabilities	Technological Capabilities	Operational Capabilities	Technological Capabilities
II_q2s6	0.782		0.788	
II_q2s7	0.706		0.722	
II_q2s8	0.813		0.765	
V_q2s29	0.526		0.520	
V_q2s30		0.798		0.787
V_q2s31		0.835		0.840
IV_q2s22		0.745		0.712
IV_q2s23	0.431	0.343		

**Table 9:** Factor analysis results for variables IV-q2s22, V-q2s29, V-q2s30, V-q2s31, II-q2s6, II-q2s7, and II-q2s8. Extraction method: principal axis factoring.

Factor	Initial Eigenvalues			Extraction Sums of Squared Loadings			Rotation Sums of Squared Loadings
	Total	% of Variance	Cumulative %	Total	% of Variance	Cumulative %	Total
1	3.798	54.259	54.259	3.384	48.346	48.346	2.950
2	1.039	14.838	69.098	0.631	9.014	57.359	2.867
3	0.607	8.671	77.769				
4	0.476	6.796	84.564				
5	0.393	5.617	90.182				
6	0.362	5.172	95.354				
7	0.325	4.646	100.000				

**Table 10:** CFA model validity measures.

Factor	CR	AVE	SQR(AVE)	Correlation between Factors
Operational capabilities	0.813	0.525	0.725	0.697
Technological capabilities	0.835	0.627	0.792	

**Table 11:** Standardized and unstandardized model paths loadings.

Relationship between Variables			Unstandardized Estimate	S.E.	C.R.	*p*	Standardized Estimate
Technological_capabilities	<---	Operational_capabilities	0.825	0.078	10.599	***	0.697
II_q2s6	<---	Operational_capabilities	1.000				0.779
II_q2s7	<---	Operational_capabilities	1.062	0.071	14.858	***	0.818
II_q2s8	<---	Operational_capabilities	0.786	0.062	12.570	***	0.681
V_q2s29	<---	Operational_capabilities	0.781	0.071	10.998	***	0.600
V_q2s30	<---	Technological_capabilities	1.000				0.785
V_q2s31	<---	Technological_capabilities	1.057	0.072	14.739	***	0.796
IV_q2s22	<---	Technological_capabilities	1.007	0.068	14.722	***	0.795

*** means that the value is smaller than 0.0001.

**Table 12:** Model fit indices.

Measure	Estimate	Threshold
CMIN	18.806	
DF	13.000	
CMIN/DF	1.447	Between 1 and 3
CFI	0.995	>0.95
SRMR	0.031	<0.08
RMSEA	0.035	<0.06
PClose	0.750	>0.05

## Data Availability

Data are contained within the article.

## References

[1] Yang J., Xie H., Liu H., Duan H. (2018). Leveraging informational and relational capabilities for performance: An empirical investigation. Int. J. Logist. Manag..

[2] Pham T.S.H., Le Monkhouse L., Barnes B.R. (2017). The influence of relational capability and marketing capabilities on the export performance of emerging market firms. Int. Mark. Rev..

[3] Ahmad N., Othman S.N., Mad Lazim H. (2014). A review of technological capability and performance relationship in manufacturing companies. Proceedings of the 2014 International Symposium on Technology Management and Emerging Technologies.

[4] Hollander J.E., Carr B.G. (2020). Virtually Perfect? Telemedicine for Covid-19. N. Engl. J. Med..

[5] Favela J., Martinez A.I., Rodriguez M.D., Gonzalez V.M. (2008). Ambient Computing Research for Healthcare: Challenges, Opportunities and Experiences. Comput. Sist..

[6] Rezai-Rad M., Vaezi R., Nattagh F. (2012). E-Health Readiness Assessment Framework in Iran. Iran. J. Public Health.

[7] Greenhalgh T., Wherton J., Papoutsi C., Lynch J., Hughes G., A’Court C., Hinder S., Fahy N., Procter R., Shaw S. (2017). Beyond adoption: A new framework for theorizing and evaluating nonadoption, abandonment, and challenges to the scale-up, spread, and sustainability of health and care technologies. J. Med. Internet Res..

[8] Weiner B.J. (2009). A theory of organizational readiness for change. Implement. Sci..

[9] Weiner B.J., Amick H., Lee S.Y.D. (2008). Review: Conceptualization and measurement of organizational readiness for change. A review of the literature in health services research and other fields. Med. Care Res. Rev..

[10] Rossmann A., Reutlingen H. (2018). Digital Maturity: Conceptualization and Measurement Model Social media View project Startups in cooperation with incumbent firms View project Digital Maturity: Conceptualization and Measurement Model. Proceedings of the Twenty-Sixth European Conference on Information Systems (ECIS 2018).

[11] Snyder-Halpern R. (2001). Indicators of organizational readiness for clinical information technology/systems innovation: A Delphi study. Int. J. Med. Inform..

[12] Jennett P., Yeo M., Pauls M., Graham J. (2003). Organizational readiness for telemedicine: Implications for success and failure. J. Telemed. Telecare.

[13] Coombs J.E., Bierly P.E. (2006). Measuring technological capability and performance. R D Manag..

[14] Christensen C.M., Overdorf M. (2000). Meeting the challenge of disruptive change. Harv. Bus. Rev..

[15] Guan J., Ma N. (2003). Innovative capability and export performance of Chinese firms. Technovation.

[16] Lu Y., Ramamurthy K. (2011). Understanding the link between information technology capability and organizational agility: An empirical examination. MIS Q. Manag. Inf. Syst..

[17] Yeniyurt S., Wu F., Kim D., Cavusgil S.T. (2019). Information technology resources, innovativeness, and supply chain capabilities as drivers of business performance: A retrospective and future research directions. Ind. Mark. Manag..

[18] Panda H., Ramanathan K. (1996). Technological capability assessment of a firm in the electricity sector. Technovation.

[19] Fortuin F.T.J.M., Omta S.W.F. (2009). Innovation drivers and barriers in food processing. Br. Food J..

[20] Ali Z., Zwetsloot I.M., Nada N. (2019). An empirical study to explore the interplay of Managerial and Operational capabilities to infuse organizational innovation in SMEs. Procedia Comput. Sci..

[21] Jonker M., Romijn H., Szirmai A. (2006). Technological effort, technological capabilities and economic performance: A case study of the paper manufacturing sector in West Java. Technovation.

[22] Mohamad A.A., Ramayah T., Lo M.C. (2017). Knowledge management in MSC Malaysia: The role of information technology capability. Int. J. Bus. Soc..

[23] Tremblay P.J. (1998). Technological Capability and Productivity Growth: An Industrialized/Industrializing Country Comparison.

[24] Ziggers G.W., Henseler J. (2009). Inter-firm network capability: How it affects buyer-supplier performance. Br. Food J..

[25] Li T., Chan Y.E. (2019). Dynamic information technology capability: Concept definition and framework development. J. Strateg. Inf. Syst..

[26] Teece D.J. (2007). Explicating dynamic capabilities: The nature and microfoundations of (sustainable) enterprise performance. Strateg. Manag. J..

[27] Avital M., Te’Eni D. (2009). From generative fit to generative capacity: Exploring an emerging dimension of information systems design and task performance. Inf. Syst. J..

[28] Doherty N.F., Terry M. (2009). The role of IS capabilities in delivering sustainable improvements to competitive positioning. J. Strateg. Inf. Syst..

[29] De Carolis A., Macchi M., Negri E., Terzi S. (2017). A.; Macchi, M.; Negri, E.; Terzi, S. A maturity model for assessing the digital readiness of manufacturing companies. IFIP Advances in Information and Communication Technology.

[30] Yusif S., Hafeez-Baig A., Soar J. (2017). e-Health readiness assessment factors and measuring tools: A systematic review. Int. J. Med. Inform..

[31] Lehman W.E.K., Greener J.M., Simpson D.D. (2002). Assessing organizational readiness for change. J. Subst. Abuse Treat..

[32] Molla A., Licker P.S. (2005). Perceived e-readiness factors in e-commerce adoption: An empirical investigation in a developing country. Int. J. Electron. Commer..

[33] Kwahk K.-Y., Lee J.-N. (2008). The role of readiness for change in ERP implementation: Theoretical bases and empirical validation. Inf. Manag..

[34] Shea C.M., Jacobs S.R., Esserman D.A., Bruce K., Weiner B.J. (2014). Organizational readiness for implementing change: A psychometric assessment of a new measure. Implement. Sci..

[35] Wiesboeck F. Thinking outside of the IT capability box. Proceedings of the Americas Conference on Information Systems 2018: Digital Disruption, AMCIS 2018.

[36] Sinha A., Kumar P., Rana N.P., Islam R., Dwivedi Y.K. (2019). Impact of internet of things (IoT) in disaster management: A task-technology fit perspective. Ann. Oper. Res..

[37] Datta P., Nwankpa J.K. (2021). Digital transformation and the COVID-19 crisis continuity planning. J. Inf. Technol. Teach. Cases.

[38] De Mori C., Batalha M.O., Alfranca O. (2016). A model for measuring technology capability in the agrifood industry companies. Br. Food J..

[39] Wang N., Liang H., Zhong W., Xue Y., Xiao J. (2014). Resource Structuring or Capability Building? An Empirical Study of the Business Value of Information Technology. J. Manag. Inf. Syst..

[40] Wang Y., Lo H., Zhang Q., Xue Y. (2006). How technological capability influences business performance: An integrated framework based on the contingency approach. J. Technol. Manag. China.

[41] Zawislak P.A., Alves A.C., Tello-Gamarra J., Barbieux D., Reichert F.M. (2012). Innovation Capability: From Technology Development to Transaction Capability. J. Technol. Manag. Innov..

[42] Zawislak P., Alves A., Tello-Gamarra J., Barbieux D., Reichert F. (2013). Influences of the internal capabilities of firms on their innovation performance: A case study investigation in Brazil. Int. J. Manag..

[43] Ali O., Shrestha A., Soar J., Wamba S.F. (2018). Cloud computing-enabled healthcare opportunities, issues, and applications: A systematic review. Int. J. Inf. Manag..

[44] Lu I.Y., Chen C.B., Wang C.H. (2007). Fuzzy multiattribute analysis for evaluating firm technological innovation capability. Int. J. Technol. Manag..

[45] Wu S.J., Melnyk S.A., Flynn B.B. (2010). Operational Capabilities: The Secret Ingredient. Decis. Sci..

[46] Benitez J., Chen Y., Teo T.S.H., Ajamieh A. (2018). Evolution of the impact of e-business technology on operational competence and firm profitability: A panel data investigation. Inf. Manag..

[47] Ahmed M.U., Kristal M.M., Pagell M. (2014). Impact of operational and marketing capabilities on firm performance: Evidence from economic growth and downturns. Int. J. Prod. Econ..

[48] Braojos J., Benitez J., Llorens J., Ruiz L. (2020). Impact of IT integration on the firm’s knowledge absorption and desorption. Inf. Manag..

[49] Raphael A., Schoemaker P.J. (1986). Strategic assets and organizational rent. Strateg. Manag. J..

[50] Helfat C.E., Peteraf M.A. (2003). The dynamic resource-based view: Capability lifecycles. Strateg. Manag. J..

[51] Benitez J., Llorens J., Braojos J. (2018). How information technology influences opportunity exploration and exploitation firm’s capabilities. Inf. Manag..

[52] Bustinza O.F., Molina L.M., Arias-Ar D. (2010). Organizational learning and performance: Relationship between the dynamic and the operational capabilities of the firm. Afr. J. Bus. Manag..

[53] Kumar P., Singh A.P. (2020). Flexibility in service operations: Review, synthesis and research agenda. Benchmarking.

[54] Ruiz-Jiménez J.M., del Fuentes-Fuentes M.M. (2016). Management capabilities, innovation, and gender diversity in the top management team: An empirical analysis in technology-based SMEs. BRQ Bus. Res. Q..

[55] Ukko J., Nasiri M., Saunila M., Rantala T. (2019). Sustainability strategy as a moderator in the relationship between digital business strategy and financial performance. J. Clean. Prod..

[56] Hair J.F.J., Black W.C., Babin B.J., Anderson R.E. (2013). Multivariate Data Analysis.

[57] GUS (2020). Główny Urząd Statystyczny, Apteki i Punkty Apteczne w 2020 r.

[58] Krejcie R.V., Morgan D.W. (1996). Determining sample Size for Research Activities. Educational and Psychological Measurement. Int. J. Employ. Stud..

[59] Tabachnick B., Fidell L. (2018). Using Multivariate Statistics.

[60] Horn J.L. (1965). Factors in Factor Analysis. Psychometrika.

[61] Bergek A., Tell F., Berggren C., Watson J. (2008). Technological capabilities and late shakeouts: Industrial dynamics in the advanced gas turbine industry, 1987–2002. Ind. Corp. Chang..

[62] Kludacz-Alessandri M., Walczak R., Hawrysz L., Korneta P. (2021). The Quality of Medical Care in the Conditions of the COVID-19 Pandemic, with Particular Emphasis on the Access to Primary Healthcare and the Effectiveness of Treatment in Poland. J. Clin. Med..

[63] Mcdermott A.M., Conway E., Rousseau D.M., Flood P.C. (2013). Promoting Effective Psychological Contracts Through Leadership: The Missing Link Between HR Strategy and Performance. Hum. Resour. Manag..

[64] Andrews R., Beynon M.J., McDermott A.M. (2016). Organizational Capability in the Public Sector: A Configurational Approach. J. Public Adm. Res. Theory.

[65] Spaulding A., Kash B.A., Johnson C.E., Gamm L. (2017). Organizational capacity for change in health care: Development and validation of a scale. Health Care Manag. Rev..

[66] Otto L., Harst L. Investigating barriers for the implementation of telemedicine initiatives: A systematic review of reviews. Proceedings of the Twenty-fifth Americas Conference on Information Systems.

[67] Hage E., Roo J.P., Van Offenbeek M.A.G., Boonstra A. (2013). Implementation factors and their effect on e-Health service adoption in rural communities: A systematic literature review. BMC Health Serv. Res..

[68] Jang-Jaccard J., Nepal S., Alem L., Li J. (2014). Barriers for Delivering Telehealth in Rural Australia: A Review Based on Australian Trials and Studies. Telemed. e-Health.

[69] Saliba V., Legido-Quigley H., Hallik R., Aaviksoo A., Car J., McKee M. (2012). Telemedicine across borders: A systematic review of factors that hinder or support implementation. Int. J. Med. Inform..

[70] Hötker E.D.V., Ring M.M., Steinhäuser J. (2021). Determinants of the Implementation of Telemedicine in the German Navy—A Mixed Methods Study. Mil. Med..

[71] Govender S.M., Mars M. (2017). The use of telehealth services to facilitate audiological management for children: A scoping review and content analysis. J. Telemed. Telecare.

[72] Gros D.F., Morland L.A., Greene C.J., Acierno R., Strachan M., Egede L.E., Tuerk P.W., Myrick H., Frueh B.C. (2013). Delivery of Evidence-Based Psychotherapy via Video Telehealth. J. Psychopathol. Behav. Assess..

[73] Kruse C.S., Bouffard S., Dougherty M., Parro J.S. (2016). Telemedicine Use in Rural Native American Communities in the Era of the ACA: A Systematic Literature Review. J. Med. Syst..

[74] Xu R., Song X., Liu G. The role of organizational capability on technological innovation. Proceedings of the 2008 ISECS International Colloquium on Computing, Communication, Control, and Management.

[75] Mamonov S., Peterson R. The Role of IT in Innovation at the Organizational Level—A Literature Review. Proceedings of the 53rd Hawaii International Conference on System Sciences.

[76] Zhang C., Dhaliwal J. (2009). An investigation of resource-based and institutional theoretic factors in technology adoption for operations and supply chain management. Int. J. Prod. Econ..

[77] Baark E., Antonio K., Lo W. Sharif Innovation sources, capabilities and competitiveness: Evidence from Hong Kong firms. Proceedings of the DIME Final Conference.

[78] Benitez-Amado J., Walczuch R.M. (2012). Information technology, the organizational capability of proactive corporate environmental strategy and firm performance: A resource-based analysis. Eur. J. Inf. Syst..

[79] Liu H., Ke W., Wei K.K., Hua Z. (2013). The impact of IT capabilities on firm performance: The mediating roles of absorptive capacity and supply chain agility. Decis. Support. Syst..

[80] Kang T., Chen H.-C., Sun J. (2016). Does Organizational Structure Influence IT Investment and its Effects on Operational Capability?. J. Res. Bus. Econ. Manag..

[81] Song M., Nason R.W., Di Benedetto C.A. (2008). Distinctive Marketing and Information Technology Capabilities and Strategic Types: A Cross-National Investigation. J. Int. Mark..

[82] Kahli R., Grover V. (2008). Business Value of IT: An Essay on Expanding Research Directions to Keep up with the Times. J. Assoc. Inf. Syst..

[83] Schryen G. (2012). Revisiting IS business value research: What we already know, what we still need to know, and how we can get there. Eur. J. Inf. Syst..

[84] Nevo S., Wade M. (2011). Firm-level benefits of IT-enabled resources: A conceptual extension and an empirical assessment. J. Strateg. Inf. Syst..

[85] Salas-Vallina A., López-Cabrales Á., Alegre J., Fernández R. (2017). On the road to happiness at work (HAW): Transformational leadership and organizational learning capability as drivers of HAW in a healthcare context. Pers. Rev..

[86] Floyd S.W., Wooldridge B. (2015). Path Analysis of the Relationship between Competitive Strategy, Information Technology, and Financial Performance. J. Manag. Inf. Syst..

